# RANTES/CCL5 and Risk for Coronary Events: Results from the MONICA/KORA Augsburg Case-Cohort, Athero-Express and CARDIoGRAM Studies

**DOI:** 10.1371/journal.pone.0025734

**Published:** 2011-12-06

**Authors:** Christian Herder, Wouter Peeters, Thomas Illig, Jens Baumert, Dominique P. V. de Kleijn, Frans L. Moll, Ulrike Poschen, Norman Klopp, Martina Müller-Nurasyid, Michael Roden, Michael Preuss, CARDIoGRAM Consortium, Mahir Karakas, Christa Meisinger, Barbara Thorand, Gerard Pasterkamp, Wolfgang Koenig

**Affiliations:** 1 Institute for Clinical Diabetology, German Diabetes Center, Leibniz Center for Diabetes Research at Heinrich Heine University Düsseldorf, Düsseldorf, Germany; 2 Department of Vascular Surgery, University Medical Center Utrecht, Utrecht, The Netherlands; 3 Research Unit of Molecular Epidemiology, Helmholtz Zentrum München, German Research Center for Environmental Health, Neuherberg, Germany; 4 Institute of Epidemiology II, Helmholtz Zentrum München, German Research Center for Environmental Health, Neuherberg, Germany; 5 Experimental Cardiology Laboratory, University Medical Center Utrecht, Utrecht, The Netherlands; 6 Interuniversity Cardiology Institute of the Netherlands, Utrecht, The Netherlands; 7 Department of Medicine I, University Hospital Grosshadern, Ludwig-Maximilians-Universität München, Munich, Germany; 8 Institute of Medical Informatics, Biometry and Epidemiology, Ludwig-Maximilians-Universität München, Munich, Germany; 9 Institute of Genetic Epidemiology, Helmholtz Zentrum München, German Research Center for Environmental Health, Neuherberg, Germany; 10 Department of Metabolic Diseases, Heinrich Heine University Düsseldorf, Düsseldorf, Germany; 11 Institute for Medical Biometrics and Statistics, Universität of Lübeck, Lübeck, Germany; 12 Department of Internal Medicine II-Cardiology, University of Ulm Medical Center, Ulm, Germany; Brigham & Women's Hospital, and Harvard Medical School, United States of America

## Abstract

**Background:**

The chemokine RANTES (regulated on activation, normal T-cell expressed and secreted)/CCL5 is involved in the pathogenesis of cardiovascular disease in mice, whereas less is known in humans. We hypothesised that its relevance for atherosclerosis should be reflected by associations between *CCL5* gene variants, RANTES serum concentrations and protein levels in atherosclerotic plaques and risk for coronary events.

**Methods and Findings:**

We conducted a case-cohort study within the population-based MONICA/KORA Augsburg studies. Baseline RANTES serum levels were measured in 363 individuals with incident coronary events and 1,908 non-cases (mean follow-up: 10.2±4.8 years). Cox proportional hazard models adjusting for age, sex, body mass index, metabolic factors and lifestyle factors revealed no significant association between RANTES and incident coronary events (HR [95% CI] for increasing RANTES tertiles 1.0, 1.03 [0.75–1.42] and 1.11 [0.81–1.54]). None of six *CCL5* single nucleotide polymorphisms and no common haplotype showed significant associations with coronary events. Also in the CARDIoGRAM study (>22,000 cases, >60,000 controls), none of these *CCL5* SNPs was significantly associated with coronary artery disease. In the prospective Athero-Express biobank study, RANTES plaque levels were measured in 606 atherosclerotic lesions from patients who underwent carotid endarterectomy. RANTES content in atherosclerotic plaques was positively associated with macrophage infiltration and inversely associated with plaque calcification. However, there was no significant association between RANTES content in plaques and risk for coronary events (mean follow-up 2.8±0.8 years).

**Conclusions:**

High RANTES plaque levels were associated with an unstable plaque phenotype. However, the absence of associations between (i) RANTES serum levels, (ii) *CCL5* genotypes and (iii) RANTES content in carotid plaques and either coronary artery disease or incident coronary events in our cohorts suggests that RANTES may not be a novel coronary risk biomarker. However, the potential relevance of RANTES levels in platelet-poor plasma needs to be investigated in further studies.

## Introduction

Inflammation is one of the hallmarks of atherosclerosis [1]. Macrophage and lymphocyte recruitment and expression of proinflammatory immune mediators characterise the initial stages of atherogenesis, and inflammatory mechanisms also contribute to progression of atherosclerosis and to plaque disruption at later stages of the disease [2]. Although these immune-mediated mechanisms are only partially understood, an increasing number of studies indicates that chemokines are important mediators of cardiovascular risk [3]–[6].

Chemokines are proinflammatory cytokines that recruit leukocytes to sites of tissue damage or infection [7]. An interesting candidate in this context is RANTES (regulated on activation, normal T-cell expressed and secreted), also known as CCL5 (C-C ligand 5) [8]. RANTES predominantly mediates chemotaxis and activation of T cells, but also of monocytes, granulocytes, mast cells and dendritic cells [9]–[13]. RANTES is mainly expressed by T cells, but there are other important cellular sources such as platelets, adipocytes, monocytes/macrophages and fibroblasts [14], [15]. Increased expression in adipose tissue and increased serum concentrations of RANTES are associated with obesity, type 2 diabetes and other cardiovascular risk factors [16]–[20].

Several lines of evidence indicate that RANTES plays a role in the pathogenesis of cardiovascular diseases. In mice, RANTES is expressed in atherosclerotic lesions and both RANTES antagonists and deletion of the gene encoding the RANTES receptor CCR5 can reduce the progression of atherosclerosis or early myocardial reperfusion [21]–[24]. In humans, the situation is less clear. Although RANTES expression has been shown convincingly for the various cell types in atherosclerotic plaques [reviewed in ref. 6], studies on the relevance of circulating RANTES concentrations as biomarker for cardiovascular risk are scarce. Moreover, population-based data on the ability of RANTES levels to predict coronary events are currently not available. Some reports on associations of polymorphisms in the genes encoding RANTES and CCR5 with coronary artery disease (CAD) also support the notion that RANTES plays a role in the development of cardiovascular disease [25].

We hypothesised that the relevance of RANTES in the development of atherosclerosis should be reflected by associations between *CCL5* genotypes, systemic RANTES levels as well as RANTES levels in atherosclerotic plaques and risk for coronary events. We tested the first two parts of this hypothesis by assessing the relationship between *CCL5* gene (encoding RANTES protein) variants and RANTES serum levels with cardiovascular risk in the German MONICA/KORA Augsburg case-cohort study. In addition, the association between *CCL5* genotypes and CAD was analysed in the large CARDIoGRAM study [26], [27]. For the third part of the hypothesis, we used carotid atherosclerotic plaques from the Dutch Athero-Express biobank study. Recently, we provided evidence that composition and biomarkers from carotid plaques predict cardiovascular outcomes [28]–[31]. Therefore, we investigated the associations between RANTES protein levels in plaques with histological plaque phenotypes and conducted a second prospective study to test whether local carotid RANTES plaque levels were associated with future coronary events.

## Materials and Methods

### MONICA/KORA Augsburg case-cohort study: design, population and follow-up

This study is a prospective case-cohort study [32] within the population-based MONICA/KORA Augsburg studies [33]–[35]. Data were derived from three independent cross-sectional, population-based surveys within the MONICA project in 1984/1985 (S1), 1989/1990 (S2) and 1994/1995 (S3) in Augsburg (Germany) and two adjacent counties. The studies were approved by the local authorities and performed according to the Declaration of Helsinki. The case-cohort study was approved by the Bayerische Landesärztekammer (Bavarian Medical Association). All participants provided written informed consent.

Response rates for the different surveys were 75.7% (S1), 74.4% (S2) and 73.1% (S3). The total number of study participants was 13,427 (6725 men, 6702 women) aged 25–64 (S1) or 25–74 years (S2, S3). All subjects were prospectively followed within the KORA framework until 2002.

The outcome variable “coronary events” was defined as combined endpoint that consisted of incident non-fatal myocardial infarction (MI) and fatal coronary death or sudden cardiac death before the age of 75 years. Cases were identified through the MONICA/KORA Augsburg coronary event registry. In addition, follow-up questionnaires were sent to persons who had moved out of the study area. Until December 2000, a major non-fatal MI was diagnosed based on the MONICA algorithm (symptoms, cardiac enzymes, ECG changes). Since January 2001 myocardial infarction was diagnosed according to criteria defined by the European Society of Cardiology (ESC) and the American College of Cardiology (ACC) [36], [37]. Coronary deaths were validated by autopsy reports, death certificates, chart review and information from the last treating physician.

Due to the low incidence of coronary events in individuals below 35 years of age, we limited the present study to persons aged 35–74 years at baseline (5382 men, 5336 women). Due to missing blood samples, 1187 subjects had to be excluded which led to a total source population of 9531 subjects (4696 men, 4835 women). Participants with self-reported prevalent coronary heart disease were also excluded, leading to a final source population of 9300 subjects (4507 men, 4793 women). For the case-cohort study, we selected a random sample of the source population (called subcohort) stratifying by sex and survey. The final stratum-specific sample sizes of this subcohort were used together with the stratum-specific sizes of the source population to compute sampling fractions, and the inverse of the sampling fractions yielded the survey and sex-specific sampling weights. All cross-sectional analyses were performed in the subcohort which included 2077 subjects (1112 men, 965 women) after exclusion of subjects with missing values for classical cardiovascular risk factors and essential inflammatory markers. Until December 31, 2002, 397 study participants had a coronary event according to our endpoint definition (307 men, 90 women). After further exclusion of subjects with incomplete information on relevant variables, the present study was based on 2271 participants (280 men, 83 women with incident coronary events; 976 men, 932 women who remained event-free). The majority of cases (n = 318, 87.6%) occurred before the end of 2000 when the diagnosis criteria were changed from the MONICA algorithm to the ESC/ACC criteria. Cases with incident coronary events included 175 participants with non-fatal MI and 188 cases with fatal coronary death or sudden cardiac death. The mean follow-up time (± standard deviation) was 10.2±4.8 years.

Sociodemographic and lifestyle variables as well as data on parental history of myocardial infarction were collected through standardised interviews. Standardised medical examinations including collection of a non-fasting venous blood sample for the determination of biomarkers were performed. All procedures and routine laboratory methods have been described in detail elsewhere [33]–[35].

### MONICA/KORA Augsburg case-cohort study: laboratory measurements

Serum levels of RANTES were determined using the Human CCL5/RANTES Quantikine ELISA kit from R&D Systems (Wiesbaden, Germany) as described [38]. Mean intra-/inter-assay coefficients of variation were 4.2% and 5.1%, respectively, for recombinant controls, and 4.1% and 6.4%, respectively, for test sera.

Six single nucleotide polymorphisms (SNPs) were selected on the basis of data from the International HapMap project (http://www.hapmap.org) to obtain complete coverage of the *CCL5* gene for a 9.2-kb locus of the *CCL5* gene (promoter: rs2107538 [−403G>A], rs2280788 [−28C>G]; intronic: rs2280789, rs4796120, rs3817655; 3′-flanking region: rs1065341). All SNPs were in Hardy-Weinberg equilibrium (p>0.2). Genotyping was carried out with matrix-assisted laser desorption ionisation-time of flight analysis of allele-dependent primer extension products as reported [39]. A subset of samples (13.3%) was genotyped in duplicate to assess genotyping quality. The mean discordance rate for the six SNPs was 0.44%.

### MONICA/KORA Augsburg case-cohort study: statistical analysis

Baseline continuous characteristics of the study participants were described using the SAS procedure SURVEYMEANS which estimated standard errors appropriate to the sampling scheme. Categorical variables and continuous variables were compared between cases and non-cases by Wald chi-square test (SURVEYFREQ) and t tests on regression coefficients (SURVEYREG), respectively. In case of non-normality, t tests were carried out for log-transformed variables, and results were given as geometric means with antilogs of standard errors of the adjusted log-means.

The associations of *CCL5* genotypes and RANTES serum levels with incident coronary events were assessed by Cox proportional hazard models. The proportional hazards assumptions for the Cox models were tested for all models graphically before the analyses and demonstrated that proportional hazards can be assumed. The case-cohort design required correction of the variance estimation based on the sampling weights to give standard error estimates for the parameter estimates. Correction for standard errors was made using the method by Barlow [32]. The inverse of the sample sizes for the subcohort by the cohort of interest yielded survey and sex-specific sampling weights. If required we additionally differentiated between cases and non-cases. Sex-specific tertiles of the weighted distributions of RANTES serum levels in the subcohort were used. Tests for trend were conducted assigning the median value within each tertile to the corresponding tertile and including this variable in the Cox regression model. A p value of <0.05 was considered to be statistically significant for the analysis of RANTES serum levels. For analyses regarding the 6 *CCL5* genotypes, the significance level was adjusted for multiple testing according to Bonferroni to 0.05/6 = 0.0083 for each SNP. All calculations were performed with SAS (Version 9.1, SAS Institute Inc., Cary, NC, USA).

### CARDIoGRAM study

The transatlantic CARDIoGRAM (Coronary ARtery DIsease Genome-wide Replication And Meta-analysis) Consortium combines data from 14 genome-wide association studies (GWAS) comprising >22,000 cases with CAD and >60,000 controls, all of European ancestry [26], [27]. Cases and controls participated in case-control or prospective cohort studies, and the case definition in the participating studies comprised CAD, MI or both (see ref. 26 for a detailed list of studies and case/control definitions). Genotype data were available from measurements on Affymetrix and Illumina platforms with subsequent imputation of genotypes in most studies [26]. The associations of *CCL5* alleles with CAD were assessed by calculating unadjusted odds ratios (OR) with corresponding 95% CI and p values per specified allele. The nominal significance level was adjusted for multiple testing according to Bonferroni to 0.05/6 = 0.0083 for each SNP in this study. The level of genome-wide significance that corrected for all tests performed in the GWAS was set at 5×10^−8^.

### Athero-Express biobank study: design, population and follow-up

The Athero-Express study is an ongoing biobank study with a longitudinal study design, comprising atherosclerotic plaques obtained from patients who underwent carotid endarterectomy (CEA) as described [40]. A multidisciplinary vascular team reviewed all indications for surgery. For the present study we included 606 atherosclerotic plaques from patients operated between 24 March 2002 and 15 December 2006. The study design has been reported previously [41]. All patients completed questionnaires including medical history and medication use prior to the operation. Patients who suffered from stroke, transient ischemic attack (TIA) or amaurosis fugax (AFX) were considered as symptomatic. Patients operated due to a significant stenosis of more than 70% and without neurological manifestations were considered as being asymptomatic. Hypertension and hypercholesterolaemia were defined as use of anti-hypertensive drugs (diuretics, beta blockers, calcium antagonists, ACE inhibitors, angiotensin II antagonists) or cholesterol-lowering drugs (statins, fibrates, acipimox, ezetimibe, colestyramine), respectively. After surgery, patients were clinically followed for up to 3 years. The composite endpoint was defined as fatal MI, sudden death, non-fatal MI and coronary intervention (including coronary bypass and coronary angioplasty). The medical ethics committees of the participating hospitals (University Medical Center Utrecht, Utrecht, and St Antonius Hospital Nieuwegein, The Netherlands) approved the study, and all participants provided written informed consent.

The collected atherosclerotic plaques were processed according to a standardised protocol [41]. Briefly, plaques were cut into small segments of 5 millimeters along the longitudinal axis. The segment with the greatest burden was considered the culprit lesion, which was subjected to histological assessment. Macrophage infiltration (CD68) and smooth muscle cell infiltration (α-actin) were quantified and expressed as percentage staining of the plaque area using computerised analyses. In addition, calcification (haematoxylin and eosin (H&E)), collagen content (Sirius Red) as well as the macrophage and smooth muscle cell stainings were scored semiquantitatively as “no”, “minor”, “moderate” or “heavy” staining [41]. The size of the lipid core (H&E and Sirius Red) was visually estimated as the percentage of the total plaque area, and plaque haemorrhage (fibrin) was scored as being present or absent. Two independent observers performed the histological examinations. The histological assessments showed good interobserver reproducibility and the semi-quantitative examinations correlated excellently with the computer-based assessments [42].

### Athero-Express biobank study: laboratory measurements

The segments adjacent to the culprit plaque lesions were used for protein isolation. Segments were dissolved in Tris buffer according to a standardised protocol and quantified for the total amount of protein [41]. RANTES levels were quantified by a multiplex assay from Bender MedSystems (Vienna, Austria) according to the manufacturer's protocol. RANTES levels were corrected for total amount of protein within the segment.

### Athero-Express biobank study: statistical analysis

Baseline RANTES levels in plaques were statistically analysed by the non-parametric Mann Whitney-U test. The association of RANTES expression with categorised histological plaque characteristics was assessed using Spearman's correlation test. The association between plaque haemorrhage and RANTES expression was determined by the Mann Whitney U test.

We used unadjusted Cox regression analyses to determine the association of RANTES plaque levels at baseline with cardiac outcome during the follow-up. In order to exclude potential confounding with respect to the association of RANTES plaque levels and cardiac endpoint, we performed multivariate analyses including all determinants that were significantly associated with RANTES expression levels at baseline. The statistical analyses were performed using SPSS 15.0 (SPSS Inc, Chicago, IL, USA).

## Results

### MONICA/KORA Augsburg case-cohort study

Individuals who experienced a coronary event during the follow-up (cases) differed in most baseline characteristics from individuals without incident coronary events (non-cases). Cases were older, had a higher BMI, higher waist-to-hip ratio, higher blood pressure, more frequently a history of hypertension or diabetes, higher total and LDL cholesterol levels, lower HDL cholesterol levels, higher ratio of total to HDL cholesterol, higher systemic concentrations of CRP and IL-6, and were less frequently physically active. These data have been reported before [43] and are given in the supporting information (Table S1).

RANTES serum levels did not differ significantly between cases and non-cases (mean 29.6 vs 28.0 ng/ml, p = 0.085). Cox proportional hazard models showed that increasing tertiles of RANTES serum concentrations were neither associated with increased risk of coronary events after adjustment for age, sex and survey (Table 1, model 1) nor after additional adjustment for anthropometric, metabolic, immunological and lifestyle factors (Table 1, models 2 and 3).

**Table 1:** Risk of coronary events according to baseline serum concentrations of RANTES (MONICA/KORA Augsburg case-cohort study).

	HR (95% CI) for tertiles of RANTES (ng/ml)*			
	Tertile 1	Tertile 2	Tertile 3	p (trend)
Median [lower-upper limit] (men)	13.1 [2.7–17.7]	22.8 [17.7–29.2]	40.4 [29.2–176.0]	
Median [lower-upper limit] (women)	13.6 [5.8–18.2]	24.5 [18.2–33.6]	46.4 [33.6–161.0]	
Number cases/non-cases	90/574	131/648	142/686	
Model 1	1.0	1.11 (0.82; 1.50)	1.24 (0.92; 1.68)	0.18
Model 2	1.0	1.05 (0.76; 1.44)	1.15 (0.83; 1.58)	0.43
Model 3	1.0	1.03 (0.75; 1.42)	1.11 (0.81; 1.54)	0.53

*Hazard ratios (HR) and 95% confidence intervals (CI) were estimated by Cox proportional hazard models. Models contained continuous variables unless otherwise indicated.

Model 1: adjusted for age, sex and survey; model 2: adjusted for factors in model 1+BMI, systolic blood pressure, ratio of total cholesterol/HDL cholesterol, history of diabetes, smoking status (never smoker, former smoker, current smoker), alcohol consumption (0, 0.1–39.9, ≥40 g/d for men; 0, 0.1–19.9, ≥20 g/d for women), physical activity (inactive, active) and parental history of myocardial infarction (negative, positive, unknown); model 3: adjusted for factors in model 2+CRP and IL-6.

In order to assess whether RANTES serum levels may be more informative for a shorter follow-up period, we repeated the analysis for coronary events occurring in the first three years (84 cases, 2187 non-cases) or in the first five years (156 cases, 2115 non-cases). HRs (95% CIs) for increasing tertiles were 1.0, 1.10 (0.62–1.98) and 1.03 (0.57–1.86) for a follow-up time of three years (p_trend_ = 0.90) and 1.0, 1.04 (0.67–1.61) and 1.06 (0.68–1.66) for a follow-up time of five years (p_trend_ = 0.88) and thus very similar to results obtained from the complete follow-up period.

Since we used non-fasting samples, we investigated the potential impact of the duration of fasting on RANTES levels. Mean duration of fasting did not differ between cases and non-cases, RANTES levels did not correlate significantly with duration of fasting in the subcohort (analysis adjusted for age, sex and survey), and additional adjustment for this variable in model 3 had no impact on HRs (data not shown).

Carriers of the minor alleles of the *CCL5* SNPs rs2107538, rs2280789, rs47966120 and rs3817655 had significantly lower RANTES serum levels (p_additive_≤3.1×10^−8^) than carriers of the major alleles in the MONICA/KORA subcohort, whereas no differences were found for rs2280788 and rs1065341 as reported previously [38]. In line with the data for serum levels, individuals who were heterozygous or homozygous for the minor alleles of the six SNPs investigated here had no decreased risk for coronary events compared to study participants who were homozygous for the respective major alleles (Table 2).

**Table 2:** Frequencies of *CCL5* genotypes in the subcohort and hazard ratios (HR) and 95% confidence intervals (CI) for incident coronary events comparing *CCL5* genotypes in the case-cohort dataset (MONICA/KORA Augsburg case-cohort study).

SNP	Allele*	n*	Weighted frequency (%)*	Model**	HR (95% CI) for genotype*		
	1/2	11/12/22	11/12/22		11	12	22
rs2107538	C/T	1382/661/76	65.2/31.2/3.6	1	1 (ref)	1.06 (0.81–1.37)	0.98 (0.52–1.84)
				2	1 (ref)	1.04 (0.79–1.37)	1.13 (0.61–2.09)
rs2280788	C/G	2036/90/0	95.8/4.2/0	1	1 (ref)	0.55 (0.26–1.13)	N/A
				2	1 (ref)	0.57 (0.26–1.23)	N/A
rs2280789	T/C	1601/488/40	75.2/22.9/1.9	1	1 (ref)	1.01 (0.76–1.33)	0.80 (0.30–2.11)
				2	1 (ref)	0.99 (0.73–1.33)	0.96 (0.39–2.40)
rs4796120	A/G	1691/414/30	79.2/19.4/1.4	1	1 (ref)	1.02 (0.68–1.53)	0.96 (0.29–3.15)
				2	1 (ref)	0.95 (0.61–1.48)	0.96 (0.29–3.15)
rs3817655	T/A	1380/665/80	64.9/31.3/3.8	1	1 (ref)	1.06 (0.81–1.37)	0.83 (0.44–1.58)
				2	1 (ref)	1.04 (0.79–1.37)	0.81 (0.40–1.65)
rs1065341	A/G	1880/231/11	88.6/10.9/0.5	1	1 (ref)	1.00 (0.74–1.34)	1.08 (0.39–2.98)
				2	1 (ref)	0.98 (0.72–1.34)	1.28 (0.50–3.31)

*Allele 1 denotes the major allele, allele 2 denotes the minor allele. All alleles are shown in forward orientation according to dbSNP BUILD 133.

**Model 1: adjusted for age, sex and survey; Model 2: Model 1+BMI, systolic blood pressure, ratio of total cholesterol / HDL cholesterol, smoking status, alcohol intake, physical activity, history of diabetes.

N/A, not applicable (no homozygous carriers of the minor allele); ref, reference genotype.

Based on the six aforementioned *CCL5* SNPs, haplotypes were constructed. None of the common haplotypes with frequencies of ≥1% in the subcohort had a statistically significant impact on the risk of coronary events compared to the most frequent haplotype CCTATA (Table 3). However, individuals who carried any of the rare haplotypes (frequencies <1% in the subcohort) had a fourfold increased risk of coronary events when these haplotypes were analysed together compared to the carriers of haplotype CCTATA (Table 3).

**Table 3:** CCL5 haplotype frequencies in the subcohort and association of haplotypes with RANTES serum levels and incident coronary events (MONICA/KORA Augsburg case-cohort study).

Haplotype*	Frequency	Association with ln (RANTES)***	Association with incident coronary events:
	(%)	*β (P)*	HR (95% CI)***
CCTATA	80.8	Reference	Reference
TCCGAA	11.0	−0.173 (4.9×10^−9^)	1.05 (0.80–1.37)
TCTAAG	5.7	−0.106 (0.0028)	1.05 (0.74–1.48)
TGCAAA	2.0	−0.073 (0.24)	0.55 (0.26–1.18)
Rare (all other)**	0.3	−0.137 (0.19)	4.04 (1.26–12.89)

*Haplotypes are based on SNPs rs2107538- rs2280788– rs2280789– rs4796120– rs3817655–rs1065341. Minor alleles of the respective SNPs are underlined.

**The category ‘rare haplotypes’ contains all haplotypes with a frequency of <1% in the subcohort.

***Adjusted for age, sex and survey. *β*, regression coefficients.

### CARDIoGRAM study

Associations between the *CCL5* SNPs rs2107538, rs2280788, rs2280789, rs4796120, 3817655 and rs1065341 and CAD were assessed in up to 82,387 individuals (>22,000 cases and >60,000 controls) in the CARDIoGRAM study (Table 4), but none of the associations reached statistical significance.

**Table 4:** Frequencies of *CCL5* alleles and allelic odds ratios (OR) and 95% confidence intervals (CI) for coronary artery disease in the CARDIoGRAM study.

SNP	n	Allele 1/2*	Frequency for allele 2 (%)	OR (95% CI) for allele 2	p
rs2107538	81,687	T / C	84.4	0.98 (0.94–1.02)	0.26
rs2280788	21,119	G / C	13.0	1.06 (0.94–1.21)	0.34
rs2280789	82,387	T / C	12.4	1.04 (0.99–1.08)	0.092
rs4796120	80,590	A / G	11.1	1.04 (0.99–1.09)	0.11
rs3817655	61,650	T / A	15.8	1.04 (1.003–1.09)	0.035
rs1065341	80,935	A / G	7.5	1.03 (0.95–1.10)	0.51

*All alleles are shown in forward orientation according to dbSNP BUILD 133.

### Athero-Express biobank study

Baseline characteristics of the Athero-Express biobank study participants and RANTES protein levels in carotid plaques stratified by clinical variables are shown in Tables 5 and 6. In an unadjusted analysis, RANTES plaque levels were lower in women compared to men and in patients with diabetes compared to non-diabetic individuals, whereas no differences were seen for other anthropometric or metabolic parameters such as BMI, hypertension or hypercholesterolaemia. Among the cardiovascular parameters, the presence of bilateral stenosis was associated with higher RANTES levels, whereas no differences could be detected for histories of vascular intervention or myocardial infarction. Increased RANTES plaque levels were also associated with increased systemic CRP levels.

**Table 5:** Baseline characteristics (demographic, anthropometric and metabolic data) related to the plaque specimens of the Athero-Express biobank study.

Patient characteristics	n (%)	RANTES (ng/µg)	*p*
		Median [IQR]	
Age (mean 68.3, SD 8.9, range 37.5–91.1 yrs)			
Sex			
Male	427 (70.5%)	46.7 [30.6–78.3]	0.05
Female	179 (29.5%)	42.7 [27.3–66.0]	
Body mass index (mean 26.4, SD 3.8, range 15.2–50.7 kg/m^2^)			
BMI<25 kg/m^2^	195 (36.8%)	45.2 [27.9–73.9]	0.21
BMI>25 kg/m^2^	335 (63.2%)	47.4 [30.6–78.3]	
Hypercholesterolaemia			
No	249 (41.2%)	46.4 [30.9–77.8]	0.49
Yes	356 (58.8%)	45.0 [28.2–74.3]	
CRP (median 3.1, IQR 1.5–6.5 mg/l)*			
<10.0 mg/l	272 (83.6%)	45.9 [30.5–75.4]	0.025
>10.0 mg/l	53 (16.3%)	58.0 [34.8–94.4]	
Current smoker			
No	433 (73.9%)	45.4 [29.1–75.2]	0.28
Yes	153 (26.1%)	47.4 [31.6–81.1]	

RANTES levels are expressed as median and interquartile range [IQR; 25^th^ percentile–75^th^ percentile].

*Systemic CRP levels were available for a subgroup of the study participants (n = 325).

**Table 6:** Baseline characteristics (clinical data and medication) related to the plaque specimens of the Athero-Express biobank study.

Patient characteristics	n (%)	RANTES (ng/µg)	*p*
		Median [IQR]	
Diabetes mellitus			
No	493 (81.5%)	47.4 [30.1–78.3]	0.03
Yes	112 (18.5%)	40.1 [28.3–60.4]	
Hypertension			
No	197 (32.6%)	45.4 [31.7–79.6]	0.22
Yes	407 (67.4%)	45.8 [27.6–73.6]	
History of vascular intervention			
No	404 (66.8%)	46.4 [30.4–75.8]	0.66
Yes	201 (33.2%)	44.6 [28.3–74.8]	
History of myocardial infarction			
No	483 (80.0%)	45.6 [29.5–76.6]	0.72
Yes	120 (20.0%)	45.5 [29.4–74.0]	
Bilateral stenosis			
No	344 (56.8%)	42.3 [26.2–67.6]	0.001
Yes	262 (43.2%)	50.6 [33.0–81.2]	
Statin use			
No	164 (27.1%)	50.2 [30.8–85.2]	0.16
Yes	441 (72.9%)	45.1 [29.3–73.3]	
Aspirin use			
No	61 (10.1%)	47.0 [27.8–82.6]	0.48
Yes	544 (89.9%)	45.5 [29.9–74.4]	
Oral anticoagulant use			
No	522 (86.3%)	45.6 [30.0–75.0]	0.88
Yes	83 (13.7%)	45.5 [28.1–80.3]	
Clinical presentation			
Asymptomatic	104 (17.2%)	48.1 [29.8–77.3]	0.63
Symptomatic (AFX, TIA or stroke)	502 (82.8%)	45.0 [29.4–75.5]	

RANTES levels are expressed as median and interquartile range [IQR; 25^th^ percentile–75^th^ percentile]. AFX, amaurosis fugax; TIA, transient ischaemic attack.

At the histological level, RANTES plaque levels were higher in plaques with less pronounced calcification (p = 0.009) and there was a trend toward higher RANTES levels in plaques with increased macrophage infiltration (p = 0.054), suggesting an association between high RANTES levels and an unstable plaque phenotype. We also found a trend for an association between RANTES levels and lipid content, but not with smooth muscle cell infiltration, collagen content, or presence of plaque haemorrhage (Table 7).

**Table 7:** RANTES expression levels (median [25^th^ percentile–75^th^ percentile] in ng/µg in relation to histological plaque composition of atherosclerotic carotid plaques (Athero-Express biobank study).

	Macrophage infiltration	Smooth muscle cell infiltration	Calcification	Collagen content	Lipid core size*	Plaque haemorrhage**
No	41.4 [29.6–61.5]	55.4 [27.3–84.0]	48.8 [32.9–79.6]	-	39.1 [24.3–65.4]	42.7 [27.5–71.2]
Minor	44.9 [27.9–71.7]	47.9 [32.2–80.0]	48.9 [29.0–78.4]	52.4 [31.6–89.0]	47.6 [30.7–82.4]	46.4 [30.0–76.6]
Moderate	44.7 [29.1–75.3]	44.9 [29.5–70.4]	48.4 [30.6–74.1]	44.9 [29.5–72.3]	46.9 [30.6–70.9]	
Heavy	58.4 [33.1–83.2]	44.5 [25.8–84.4]	38.0 [25.5–67.7]	43.7 [27.3–74.7]		
Correlation coefficient	0.08	−0.18	−0.11	−0.05	0.07	−0.96
*P*	0.054	0.66	0.009	0.24	0.09	0.34

Correlations of the histological plaque characteristics with continuous RANTES levels were analysed by Spearman's bivariate correlation test.

*Lipid core size was expressed as no, <40% and >40% of the total plaque area.

**Presence of plaque haemorrhage was scored as present or absent and statistically analysed by the Mann Whitney-U test.

**Figure 1:**
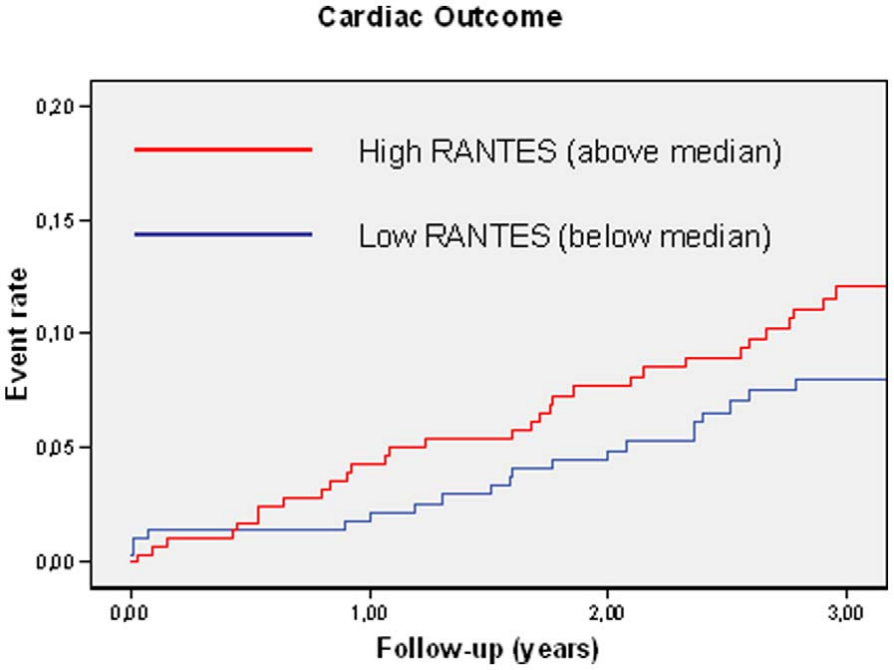
Unadjusted relationship between RANTES levels in carotid plaques and cardiac clinical outcome (Athero-Express biobank study).

During a mean follow-up of 2.8 years (SD 0.8 years, maximum 3 years), 51 study participants (8.5%) reached a cardiac endpoint (cases). These included 31 individuals who died and 20 individuals who underwent a cardiac intervention due to coronary artery disease. RANTES plaque levels did not differ between individuals without and with events (median [25^th^ percentile, 75^th^ percentile] 44.86 [29.04–74.96] vs 57.77 [34.52–79.90] ng/µg; respectively; p = 0.12). When the event rates of individuals with high (above the median of 45.52 ng/µg) and low (<45.52 ng/µg) RANTES levels in plaques were compared, unadjusted Cox regression analysis revealed no association with higher event risk for higher RANTES levels (HR [95% CI] 1.51 [0.86–2.64], p = 0.16) (Fig. 1, Table 8). Adjustment for baseline determinants of RANTES levels (i.e., sex, diabetes status, presence of bilateral stenosis, CRP) attenuated the association even further (HR [95% CI] 1.01 [0.99–1.01]; p = 0.87).

**Table 8:** Risk of coronary events according to baseline plaque levels of RANTES (Athero-Express Biobank Study).

	Univariate analysis		Multivariate analysis	
	HR (95% CI)	p	HR (95% CI)	p
RANTES (above vs below median)	1.51 (0.86–2.64)	0.16	1.01 (0.99–1.01)	0.87
Sex (men vs women)	2.55 (1.15–5.56)	0.02	2.10 (0.80–5.53)	0.13
Diabetes (yes vs no)	1.60 (0.85–3.00)	0.15	2.05 (0.87–4.82)	0.10
Bilateral stenosis (yes vs no)	1.46 (0.84–2.53)	0.18	1.61 (0.77–3.39)	0.21
CRP (mg/l)	1.01 (1.00–1.02)	0.16	1.01 (0.99–1.02)	0.22

Data are given as hazard ratios (HR) and 95% confidence intervals (CI).

## Discussion

Our study has three main findings: (i) RANTES serum levels were not associated with incident coronary events in the MONICA/KORA Augsburg case-cohort study, (ii) functional RANTES/*CCL5* SNPs were not associated with CAD in the large CARDIoGRAM study, and (iii) RANTES levels were associated with an unstable phenotype of carotid plaques, but no independent predictor of future coronary events in the Athero-Express study.

### RANTES serum levels and incident CHD

Prospective studies on systemic RANTES levels and cardiovascular outcomes are very scarce and controversial. Whereas higher 2-year cardiac mortality in male patients undergoing coronary angiography has been found associated with *reduced* levels of RANTES plasma levels [44], *elevated* RANTES levels predicted future cardiovascular events in a small sample of patients with unstable angina pectoris [45]. In patients with acute coronary syndromes RANTES plasma levels were – in contrast to concentrations of most other biomarkers measured - *not predictive* of future cardiovascular events [46]. In all three studies, the number of incident cases was substantially lower than in the MONICA/KORA Augsburg case-cohort study, and follow-up periods were shorter. Furthermore, these aforementioned studies were performed in subjects with suspected and/or preexisting cardiovascular disease, which limits the conclusions that can be drawn regarding cardiovascular risk in the general population. Therefore, our prospective MONICA/KORA Augsburg case-cohort study extends the current literature as it represents the first large, population-based study to characterise the relationship between systemic RANTES concentrations and cardiovascular outcomes.

### RANTES/*CCL5* SNPs and cardiovascular risk

In recent years, several candidate studies analysed whether *CCL5* SNPs could serve as biomarkers for cardiovascular risk. Most data are available for the *CCL5* SNP rs2107538 which is also known as −403GA. This SNP is likely to be functional as we showed in the MONICA/KORA cohort before that carriers of the minor allele had significantly lower RANTES serum levels [38]. Alleles or genotypes of rs2107538 were associated with CAD in patients who underwent coronary angiography [47], with all-cause mortality (mainly attributable to cardiac events) in patients with type 2 diabetes and end-stage renal disease [48] and with presence and severity of CAD in patients subjected to percutaneous coronary intervention [49]. In contrast, rs2107538 variants showed no associations with CAD in a case-control study [50] or with presence of significant (>50%) stenosis in patients who underwent elective coronary angiography [51]. Overall, these data were inconclusive with regard to associations between rs2107538 and cardiovascular disease, were confined to patient groups and therefore cannot be extrapolated to the general population.

Given the low statistical power of the aforementioned single studies it is of interest that associations with genome-wide significance have not been reported for SNPs in or near the *CCL5* locus in recent GWAS that covered this genomic region [27], [52], [53], [54]. However, from these reports it was not clear whether this lack of evidence was most likely due to an absence of any association or whether results for SNPs in/near this locus suggested that genome-wide significance could be reached by a reasonable increase in sample size.

The data from the MONICA/KORA study represent the first report on associations between *CCL5* genotypes and incident coronary events from a population-based study. Due to limited statistical power we sought to substantiate our findings in the CARDIoGRAM consortium, currently the largest database on gene variants and CAD worldwide. As recently published, the CARDIoGRAM study is adequately powered to reveal associations at genome-wide significance level (p<5×10^−8^) between common SNPs and CAD even for allelic OR of <1.10 [27], but associations between *CCL5* genotypes and CAD were not nominally significant and far from genome-wide significance. It should be noted that in contrast to the prospective MONICA/KORA Augsburg case-cohort that evaluated risk factors for incident coronary events the CARDIoGRAM study investigated gene variants that are associated with prevalent CAD and thus does not represent a perfect replication sample. However, we believe that any major impact of RANTES genotypes on coronary risk should also have been reflected by an association with prevalent disease so that the CARDIoGRAM data add relevant information to the initial findings from the MONICA/KORA Augsburg case-cohort study.

The MONICA/KORA Augsburg study also allowed for the evaluation of haplotypes as risk factors of coronary events. Our data indicate that rare haplotypes may indeed comprise variants that are associated with increased coronary risk. However, this study does not have the statistical power to analyse this aspect in more detail, and data on DNA sequences would be needed to identify defined haplotypes with potential relevance for pathogenesis and prediction. Therefore, this finding is hypothesis-generating at best and needs corroboration in further studies.

Taken together, our data strongly suggest that the six *CCL5* SNPs that we included in our study and the major haplotypes based on them may not be relevant biomarkers for the prediction of cardiovascular events.

### Predictive value of RANTES protein levels in plaques

Data from mouse models demonstrate that RANTES and its receptor CCR5 are expressed in atheromas, that they play a causal role in atherogenesis in these models and that interference with leukocyte migration at this level may represent a promising therapeutic approach to prevent or delay atherosclerotic processes [5]. Hence, it can be hypothesised that higher RANTES levels may be indicative of elevated cardiovascular risk.

Higher *CCL5* gene expression has indeed been reported in atherosclerotic tissue from individuals with symptomatic carotid plaques (i.e., symptoms within the past 6 months) compared to *CCL5* gene expression in iliac arteries from organ donors [55]. In contrast, several chemokines were upregulated in downstream compared to upstream portions of carotid plaques in patients undergoing carotid endarterectomy for extracranial high-grade internal carotid stenosis, but no differences in gene expression could be seen for *CCL5* [56]. In the Atherosclerosis Risk in Communities (ARIC) Carotid MRI study, RANTES plasma levels were associated with the extent of atherosclerosis and high-risk plaques, but RANTES levels in plaques were not measured in this study [57].

Our findings from the Athero-Express study extend current knowledge on RANTES plaque levels as potential biomarker because it relates RANTES plaque levels and risk for coronary events in a prospective design. Although we observed a trend toward higher risk for higher RANTES plaque levels, this association was completely abolished when we accounted for several established cardiovascular risk factors in a multivariate analysis. However, it should be noted that we found an association between RANTES and an unstable plaque phenotype which is in line with previous studies investigating the potential role of RANTES in the development of cardiovascular disease. It is noteworthy that some of the classical cardiovascular risk factors such as male sex, diabetes or systemic CRP were not significantly associated with the risk for coronary events in the multivariate model. We hypothesise that both the low number of events (compared to studies like the MONICA/KORA Augsburg case-cohort study) and the fact that the study sample represents a high-risk group that differs from the general population may have contributed to this finding.

### Strengths of our study

The combination of three independent studies to investigate different biological levels (gene variants, local and systemic protein concentrations) of RANTES in order to assess their use as potential biomarkers for the risk of coronary events represents the main strength of our study. Our findings are based on study cohorts that have been used before to identify novel associations between inflammation-related proteins (MONICA/KORA, Athero-Express), gene variants (CARDIoGRAM) and cardiovascular risk. The MONICA/KORA study is the first population-based study to examine the relationship between *CCL5* SNPs, RANTES serum levels and risk for coronary events. Its strengths include the population-based design, a long follow-up and a large number of cases. The Athero-Express study also represents the first study to test whether RANTES plaque levels predict cardiovascular outcome. The fact that the same cohort and the same methods allowed us to identify other prognostic proteins in carotid plaques such as fatty-acid binding protein, matrix metalloproteinase-8 and osteopontin as predictors of cardiovascular events underlines that the study design is indeed suitable to address our study question [29]–[31]. The CARDIoGRAM database is currently the largest database worldwide on common SNPs and CAD and thus the best available data source for our study aim.

### Study limitations

In the MONICA/KORA Augsburg case-cohort study we measured RANTES in serum samples which is important to note because RANTES is released by platelets during the clotting process. Therefore, we cannot exclude that the analysis of platelet-poor or platelet-free plasma would have led to different results. Platelet count is only available in MONICA/KORA survey 3, so that we could not adjust for this potentially relevant confounder in our analysis. A general limitation of current studies on RANTES and disease outcomes, including our own (MONICA/KORA and Athero-Express), is the fact that commercially available assays measure total RANTES protein, although variant forms including truncated proteins and proteins with different oxidation, glycation and glycosylation patterns exist. More research is needed to reveal whether these variants may be more informative in prospective studies than total RANTES levels [58]. Finally, the CARDIoGRAM consortium combined studies with different designs and outcome definitions which can be expected to attenuate associations between genetic variation and risk for coronary events.

### Conclusion

We found an association between high RANTES plaque levels and an unstable plaque phenotype, but no associations of (i) RANTES serum levels, (ii) *CCL5* genotypes and (iii) RANTES content in carotid plaques with coronary artery disease or incident coronary events in our cohorts. These data indicate that RANTES/*CCL5* gene variants and protein levels may not be novel biomarkers for the risk of coronary events in humans. However, our study could not address the questions whether measurement of RANTES in platelet-poor plasma or measurement of variant forms of RANTES proteins (locally or systemically) may be more informative for cardiovascular risk prediction than the approaches we pursued. Moreover, we cannot exclude that the measurement of RANTES may be of prognostic benefit in specific patient groups.

## Supporting Information

Text S1 **Members and affiliations of the CARDIoGRAM Consortium are listed in the Supplementary Information.**
(DOC)

Text S2 **Funding information and competing interests of the CARDIoGRAM Consortium.**
(DOC)

Table S1 **Baseline demographic, lifestyle and clinical characteristics of the study participants without and with incident coronary event during follow-up (MONICA/KORA Augsburg case-cohort study).**
(DOC)
