# Supplementary material for: RANTES/CCL5 and Risk for Coronary Events: Results from the MONICA/KORA Augsburg Case-Cohort, Athero-Express and CARDIoGRAM Studies
Source: PLoS One. 2011 Dec 6;6(12):e25734. doi: 10.1371/journal.pone.0025734 (PMC3232218; doi:10.1371/journal.pone.0025734)
Supplement: Text S1 — Members and affiliations of the CARDIoGRAM Consortium are listed in the Supplementary Information. (DOC) [file pone.0025734.s001.doc]

Online Text S1. Members and affiliations of the CARDIoGRAM Consortium.

**Executive Committee**: Sekar Kathiresan^1,2,3^, Muredach P. Reilly^4^, Nilesh J. Samani^5,6^, Heribert Schunkert^7^

**Executive Secretary:** Jeanette Erdmann^7^

**Steering Committee:** Themistocles L. Assimes^8^, Eric Boerwinkle^9^, Jeanette Erdmann^7^, Alistair Hall^10^, Christian Hengstenberg^11^, Sekar Kathiresan^1,2,3^, Inke R. König^12^, Reijo Laaksonen^13^, Ruth McPherson^14^, Muredach P. Reilly^4^, Nilesh J. Samani^5,6^, Heribert Schunkert^7^, John R. Thompson^15^, Unnur Thorsteinsdottir^16,17^, Andreas Ziegler^12^

**Statisticians**: Inke R. König^12^ (chair), John R. Thompson^15^ (chair), Devin Absher^18^, Li Chen^19^, L. Adrienne Cupples^20,21^, Eran Halperin^22^, Mingyao Li^23^, Kiran Musunuru^1,2,3^, Michael Preuss^12,7^, Arne Schillert^12^, Gudmar Thorleifsson^16^, Benjamin F. Voight^2,3,24^, George A. Wells^25^

**Writing group**: Themistocles L. Assimes^8^, Panos Deloukas^26^, Jeanette Erdmann^7^, Hilma Holm^16^, Sekar Kathiresan^1,2,3^, Inke R. König^12^, Ruth McPherson^14^, Muredach P. Reilly^4^, Robert Roberts^14^, Nilesh J. Samani^5,6^, Heribert Schunkert^7^, Alexandre F. R. Stewart^14^

**ADVANCE:** Devin Absher^18^, Themistocles L. Assimes^8^, Stephen Fortmann^8^, Alan Go^27^, Mark Hlatky^8^, Carlos Iribarren^27^, Joshua Knowles^8^, Richard Myers^18^, Thomas Quertermous^8^, Steven Sidney^27^, Neil Risch^28^, Hua Tang^29^

**CADomics**: Stefan Blankenberg^30^, Tanja Zeller^30^, Arne Schillert^12^, Philipp Wild^30^, Andreas Ziegler^12^, Renate Schnabel^30^, Christoph Sinning^30^, Karl Lackner^31^, Laurence Tiret^32^, Viviane Nicaud^32^, Francois Cambien^32^, Christoph Bickel^30^, Hans J. Rupprecht^30^, Claire Perret^32^, Carole Proust^32^, Thomas Münzel^30^

**CHARGE**: Maja Barbalic^33^, Joshua Bis^34^, Eric Boerwinkle^9^, Ida Yii-Der Chen^35^, L. Adrienne Cupples^20,21^, Abbas Dehghan^36^, Serkalem Demissie-Banjaw^37,21^, Aaron Folsom^38^, Nicole Glazer^39^, Vilmundur Gudnason^40,41^, Tamara Harris^42^, Susan Heckbert^43^, Daniel Levy^21^, Thomas Lumley^44^, Kristin Marciante^45^, Alanna Morrison^46^, Christopher J. O´Donnell^47^, Bruce M. Psaty^48^, Kenneth Rice^49^, Jerome I. Rotter^35^, David S. Siscovick^50^, Nicholas Smith^43^, Albert Smith^40,41^, Kent D. Taylor^35^, Cornelia van Duijn^36^, Kelly Volcik^46^, Jaqueline Whitteman^36^, Vasan Ramachandran^51^, Albert Hofman^36^, Andre Uitterlinden^52,36^

**deCODE**: Solveig Gretarsdottir^16^, Jeffrey R. Gulcher^16^, Hilma Holm^16^, Augustine Kong^16^, Kari Stefansson^16,17^, Gudmundur Thorgeirsson^53,17^, Karl Andersen^53,17^, Gudmar Thorleifsson^16^, Unnur Thorsteinsdottir^16,17^

**GERMIFS I and II:** Jeanette Erdmann^7^, Marcus Fischer^11^, Anika Grosshennig^12,7^, Christian Hengstenberg^11^, Inke R. König^12^, Wolfgang Lieb^54^, Patrick Linsel-Nitschke^7^, Michael Preuss^12,7^, Klaus Stark^11^, Stefan Schreiber^55^, H.-Erich Wichmann^56,58,59^, Andreas Ziegler^12^, Heribert Schunkert^7^

**GERMIFS III (KORA)**: Zouhair Aherrahrou^7^, Petra Bruse^7^, Angela Doering^56^, Jeanette Erdmann^7^, Christian Hengstenberg^11^, Thomas Illig^56^, Norman Klopp^56^, Inke R. König^12^, Patrick Linsel-Nitschke^7^, Christina Loley^12,7^, Anja Medack^7^, Christina Meisinger^56^, Thomas Meitinger^57,60^, Janja Nahrstedt^12,7^, Annette Peters^56^, Michael Preuss^12,7^, Klaus Stark^11^, Arnika K. Wagner^7^, H.-Erich Wichmann^56,58,59^, Christina Willenborg^12,7^, Andreas Ziegler^12^, Heribert Schunkert^7^

**LURIC/AtheroRemo**: Bernhard O. Böhm^61^, Harald Dobnig^62^, Tanja B. Grammer^63^, Eran Halperin^22^, Michael M. Hoffmann^64^, Marcus Kleber^65^, Reijo Laaksonen^13^, Winfried März^63,66,67^, Andreas Meinitzer^66^, Bernhard R. Winkelmann^68^, Stefan Pilz^62^, Wilfried Renner^66^, Hubert Scharnagl^66^, Tatjana Stojakovic^66^, Andreas Tomaschitz^62^, Karl Winkler^64^

**MIGen**: Benjamin F. Voight^2,3,24^, Kiran Musunuru^1,2,3^, Candace Guiducci^3^, Noel Burtt^3^, Stacey B. Gabriel^3^, David S. Siscovick^50^, Christopher J. O’Donnell^47^, Roberto Elosua^69^, Leena Peltonen^49^, Veikko Salomaa^70^, Stephen M. Schwartz^50^, Olle Melander^26^, David Altshuler^71,3^, Sekar Kathiresan^1,2,3^

**OHGS**: Alexandre F. R. Stewart^14^, Li Chen^19^, Sonny Dandona^14^, George A. Wells^25^, Olga Jarinova^14^, Ruth McPherson^14^, Robert Roberts^14^

**PennCATH/MedStar**: Muredach P. Reilly^4^, Mingyao Li^23^, Liming Qu^23^, Robert Wilensky^4^, William Matthai^4^, Hakon H. Hakonarson^72^, Joe Devaney^73^, Mary Susan Burnett^73^, Augusto D. Pichard^73^, Kenneth M. Kent^73^, Lowell Satler^73^, Joseph M. Lindsay^73^, Ron Waksman^73^, Christopher W. Knouff^74^, Dawn M. Waterworth^74^, Max C. Walker^74^, Vincent Mooser^74^, Stephen E. Epstein^73^, Daniel J. Rader^75,4^

**WTCCC**: Nilesh J. Samani^5,6^, John R. Thompson^15^, Peter S. Braund^5^, Christopher P. Nelson^5^, Benjamin J. Wright^76^, Anthony J. Balmforth^77^, Stephen G. Ball^78^, Alistair S. Hall^10^, Wellcome Trust Case Control Consortium

**Affiliations**

1 Cardiovascular Research Center and Cardiology Division, Massachusetts General Hospital, Boston, MA, USA; 2 Center for Human Genetic Research, Massachusetts General Hospital, Boston, MA, USA; 3 Program in Medical and Population Genetics, Broad Institute of Harvard and Massachusetts Institute of Technology (MIT), Cambridge, MA, USA; 4 The Cardiovascular Institute, University of Pennsylvania, Philadelphia, PA, USA; 5 Department of Cardiovascular Sciences, University of Leicester, Glenfield Hospital, Leicester, UK; 6 Leicester National Institute for Health Research Biomedical Research Unit in Cardiovascular Disease, Glenfield Hospital, Leicester, LE3 9QP, UK; 7 Medizinische Klinik II, Universität zu Lübeck, Lübeck, Germany; 8 Department of Medicine, Stanford University School of Medicine, Stanford, CA, USA; 9 University of Texas Health Science Center, Human Genetics Center and Institute of Molecular Medicine, Houston, TX, USA; 10 Division of Cardiovascular and Neuronal Remodelling, Multidisciplinary Cardiovascular Research Centre, Leeds Institute of Genetics, Health and Therapeutics, University of Leeds, UK; 11 Klinik und Poliklinik für Innere Medizin II, Universität Regensburg, Regensburg, Germany; 12 Institut für Medizinische Biometrie und Statistik, Universität zu Lübeck, Lübeck, Germany; 13 Science Center, Tampere University Hospital, Tampere, Finland; 14 The John & Jennifer Ruddy Canadian Cardiovascular Genetics Centre, University of Ottawa Heart Institute, Ottawa, Canada; 15 Department of Health Sciences, University of Leicester, Leicester, UK; 16 deCODE Genetics, 101 Reykjavik, Iceland; 17 University of Iceland, Faculty of Medicine, 101 Reykjavik, Iceland; 18 Hudson Alpha Institute, Huntsville, Alabama, USA; 19 Cardiovascular Research Methods Centre, University of Ottawa Heart Institute, 40 Ruskin Street, Ottawa, Ontario, Canada, K1Y 4W7; 20 Department of Biostatistics, Boston University School of Public Health, Boston, MA USA; 21 National Heart, Lung and Blood Institute's Framingham Heart Study, Framingham, MA, USA; 22 The Blavatnik School of Computer Science and the Department of Molecular Microbiology and Biotechnology, Tel-Aviv University, Tel-Aviv, Israel, and the International Computer Science Institute, Berkeley, CA, USA; 23 Biostatistics and Epidemiology, University of Pennsylvania, Philadelphia, PA, USA; 24 Department of Medicine, Harvard Medical School, Boston, MA, USA; 25 Research Methods, Univ Ottawa Heart Inst; 26 Department of Clinical Sciences, Hypertension and Cardiovascular Diseases, Scania University Hospital, Lund University, Malmö, Sweden; 27 Division of Research, Kaiser Permanente, Oakland, CA, USA; 28 Institute for Human Genetics, University of California, San Francisco, San Francisco, CA, USA; 29 Dept Cardiovascular Medicine, Cleveland Clinic; 30 Medizinische Klinik und Poliklinik, Johannes-Gutenberg Universität Mainz, Universitätsmedizin, Mainz, Germany; 31 Institut für Klinische Chemie und Laboratoriumsmediizin, Johannes-Gutenberg Universität Mainz, Universitätsmedizin, Mainz, Germany; 32 INSERM UMRS 937, Pierre and Marie Curie University (UPMC, Paris 6) and Medical School, Paris, France; 33 University of Texas Health Science Center, Human Genetics Center, Houston, TX, USA; 34 Cardiovascular Health Resarch Unit and Department of Medicine, University of Washington, Seattle, WA USA; 35 Cedars-Sinai Medical Center, Medical Genetics Institute, Los Angeles, CA, USA; 36 Erasmus Medical Center, Department of Epidemiology, Rotterdam, The Netherlands; 37 Boston University, School of Public Health, Boston, MA, USA; 38 University of Minnesota School of Public Health, Division of Epidemiology and Community Health, School of Public Health (A.R.F.), Minneapolis, MN, USA; 39 University of Washington, Cardiovascular Health Research Unit and Department of Medicine, Seattle, WA, USA; 40 Icelandic Heart Association, Kopavogur Iceland; 41 University of Iceland, Reykjavik, Iceland; 42 Laboratory of Epidemiology, Demography, and Biometry, Intramural Research Program, National Institute on Aging, National Institutes of Health, Bethesda MD, USA; 43 University of Washington, Department of Epidemiology, Seattle, WA, USA; 44 University of Washington, Department of Biostatistics, Seattle, WA, USA; 45 University of Washington, Department of Internal Medicine, Seattle, WA, USA; 46 University of Texas, School of Public Health, Houston, TX, USA; 47 National Heart, Lung and Blood Institute, Framingham Heart Study, Framingham, MA and Cardiology Division, Massachusetts General Hospital, Boston, MA, USA; 48 Center for Health Studies, Group Health, Departments of Medicine, Epidemiology, and Health Services, Seattle, WA, USA; 49 The Wellcome Trust Sanger Institute, The Wellcome Trust Genome Campus, Hinxton, Cambridge, UK; 50 Cardiovascular Health Research Unit, Departments of Medicine and Epidemiology, University of Washington, Seattle; 51 Boston University Medical Center, Boston, MA, USA; 52 Department of Internal Medicine, Erasmus Medical Center, Rotterdam, The Netherlands; 53 Department of Medicine, Landspitali University Hospital, 101 Reykjavik, Iceland; 54 Boston University School of Medicine, Framingham Heart Study, Framingham, MA, USA; 55 Institut für Klinische Molekularbiologie, Christian-Albrechts Universität, Kiel, Germany; 56 Institute of Epidemiology, Helmholtz Zentrum München – German Research Center for Environmental Health, Neuherberg, Germany; 57 Institut für Humangenetik, Helmholtz Zentrum München, Deutsches Forschungszentrum für Umwelt und Gesundheit, Neuherberg, Germany; 58 Institute of Medical Information Science, Biometry and Epidemiology, Ludwig-Maximilians-Universität München, Germany; 59 Klinikum Grosshadern, Munich, Germany; 60 Institut für Humangenetik, Technische Universität München, Germany; 61 Division of Endocrinology and Diabetes, Graduate School of Molecular Endocrinology and Diabetes, University of Ulm, Ulm, Germany; 62 Division of Endocrinology, Department of Medicine, Medical University of Graz, Austria; 63 Synlab Center of Laboratory Diagnostics Heidelberg, Heidelberg, Germany; 64 Division of Clinical Chemistry, Department of Medicine, Albert Ludwigs University, Freiburg, Germany; 65 LURIC non profit LLC, Freiburg, Germany; 66 Clinical Institute of Medical and Chemical Laboratory Diagnostics, Medical University Graz, Austria; 67 Institute of Public Health, Social and Preventive Medicine, Medical Faculty Manneim, University of Heidelberg, Germany; 68 Cardiology Group Frankfurt-Sachsenhausen, Frankfurt, Germany; 69 Cardiovascular Epidemiology and Genetics Group, Institut Municipal d’Investigació Mèdica, Barcelona; Ciber Epidemiología y Salud Pública (CIBERSP), Spain; 70 Chronic Disease Epidemiology and Prevention Unit, Department of Chronic Disease Prevention, National Institute for Health and Welfare, Helsinki, Finland; 71 Department of Molecular Biology and Center for Human Genetic Research, Massachusetts General Hospital, Harvard Medical School, Boston, USA; 72 The Center for Applied Genomics, Children’s Hospital of Philadelphia, Philadelphia, Pennsylvania, USA; 73 Cardiovascular Research Institute, Medstar Health Research Institute, Washington Hospital Center, Washington, DC 20010, USA; 74 Genetics Division and Drug Discovery, GlaxoSmithKline, King of Prussia, Pennsylvania 19406, USA; 75 The Institute for Translational Medicine and Therapeutics, School of Medicine, University of Pennsylvania, Philadelphia, PA, USA; 76 Department of Cardiovascular Surgery, University of Leicester, Leicester, UK; 77 Division of Cardiovascular and Diabetes Research, Multidisciplinary Cardiovascular Research Centre, Leeds Institute of Genetics, Health and Therapeutics, University of Leeds, Leeds, LS2 9JT, UK; 78 LIGHT Research Institute, Faculty of Medicine and Health, University of Leeds, Leeds, UK
