# Supplementary material for: RANTES/CCL5 and Risk for Coronary Events: Results from the MONICA/KORA Augsburg Case-Cohort, Athero-Express and CARDIoGRAM Studies
Source: PLoS One. 2011 Dec 6;6(12):e25734. doi: 10.1371/journal.pone.0025734 (PMC3232218; doi:10.1371/journal.pone.0025734)
Supplement: Text S2 — Funding information and competing interests of the CARDIoGRAM Consortium. (DOC) [file pone.0025734.s002.doc]

## Online Text S2. Funding information and competing interests of the CARDIoGRAM Consortium

The **ADVANCE** study was supported by a grant from the Reynold's Foundation and NHLBI grant HL087647.

Genetic analyses of **CADomics** were supported by a research grant from Boehringer Ingelheim. Recruitment and analysis of the CADomics cohort was supported by grants from Boehringer Ingelheim and PHILIPS medical Systems, by the Government of Rheinland-Pfalz in the context of the “Stiftung Rheinland-Pfalz für Innovation”, the research program “Wissen schafft Zukunft” and by the Johannes-Gutenberg University of Mainz in the context of the “Schwerpunkt Vaskuläre Prävention” and the “MAIFOR grant 2001”, by grants from the Fondation de France, the French Ministry of Research, and the Institut National de la Santé et de la Recherche Médicale.

The **deCODE** CAD/MI Study was sponsored by NIH grant, National Heart, Lung and Blood Institute R01HL089650-02.

The German MI Family Studies (**GerMIFS I-III (KORA)**) were supported by the Deutsche Forschungsgemeinschaft and the German Federal Ministry of Education and Research (BMBF) in the context of the German National Genome Research Network (NGFN-2 and NGFN-plus), the EU funded integrated project Cardiogenics (LSHM-CT-2006-037593), and the bi-national BMBF/ANR funded project CARDomics (01KU0908A).

**LURIC** has received funding from the EU framework 6 funded Integrated Project “Bloodomics” (LSHM-CT-2004-503485), the EU framework 7 funded Integrated Project AtheroRemo (HEALTH-F2-2008-201668) and from Sanofi/Aventis, Roche, Dade Behring/Siemens, and AstraZeneca.

The **MIGen** study was funded by the US National Institutes of Health (NIH) and National Heart, Lung, and Blood Institute’s STAMPEED genomics research program through R01 HL087676. Ron Do from the MIGen study is supported by a Canada Graduate Doctoral Scholarship from the Canadian Institutes of Health Research.

Recruitment of **PennCATH** was supported by the Cardiovascular Institute of the University of Pennsylvania. Recruitment of the **MedStar** sample was supported in part by the MedStar Research Institute and the Washington Hospital Center and a research grant from GlaxoSmithKline. Genotyping of PennCATH and Medstar was performed at the Center for Applied Genomics at the Children’s Hospital of Philadelphia and supported by GlaxoSmithKline through an Alternate Drug Discovery Initiative research alliance award (M. P. R. and D. J. R.) with the University of Pennsylvania School of Medicine.

The **Ottawa Heart Genomic Study** was supported by CIHR #MOP--82810 (R. R.), CFI #11966 (R. R.), HSFO #NA6001 (R. McP.), CIHR #MOP172605 (R. McP.), CIHR #MOP77682 (A. F. R. S.).

The **WTCCC** Study was funded by the Wellcome Trust. Recruitment of cases for the WTCCC Study was carried out by the British Heart Foundation (BHF) Family Heart Study Research Group and supported by the BHF and the UK Medical Research Council. N. J. S. and S. G. B. hold chairs funded by the British Heart Foundation. N. J. S. and A.H.G are also supported by the Leicester NIHR Biomedical Research Unit in Cardiovascular Disease and the work described in this paper is part of the research portfolio of the Leicester NIHR Biomedical Research Unit.

The **Age, Gene/Environment Susceptibility Reykjavik Study** has been funded by NIH contract N01-AG-12100, the NIA Intramural Research Program, Hjartavernd (the Icelandic Heart Association), and the Althingi (the Icelandic Parliament).

The **Cleveland Clinic GeneBank** study was supported by NIH grants P01 HL098055, P01HL076491-06, R01DK080732, P01HL087018, and 1RO1HL103931-01.

The collection of clinical and sociodemographic data in the **Dortmund Health Study** was supported by the German Migraine- & Headache Society (DMKG) and by unrestricted grants of equal share from Astra Zeneca, Berlin Chemie, Boots Healthcare, Glaxo-Smith-Kline, McNeil Pharma (former Woelm Pharma), MSD Sharp & Dohme and Pfizer to the University of Muenster. Blood collection was done through funds from  the Institute of Epidemiology and Social Medicine, University of Muenster.

The **EPIC-Norfolk study** is supported by the Medical Research Council UK and Cancer Research UK.

The **EpiDREAM study** is supported by the Canadian Institutes fo Health Research, Heart and Stroke Foundation of Ontario, Sanofi-Aventis, GlaxoSmithKline and King Pharmaceuticals.

Funding for Andrew Lotery from the **LEEDS** study was provided by tha T.F.C. Frost charity and the Macular Disease Society.

The **Rotterdam Study** is supported by the Erasmus Medical Center and Erasmus University Rotterdam; the Netherlands Organization for Scientific Research; the Netherlands Organization for Health Research and Development (ZonMw); the Research Institute for Diseases in the Elderly; The Netherlands Heart Foundation; the Ministry of Education, Culture and Science; the Ministry of Health Welfare and Sports; the European Commission (DG XII); and the Municipality of Rotterdam. Support for genotyping was provided by the Netherlands Organization for Scientific Research (NWO) (175.010.2005.011, 911.03.012), the Netherlands Genomics Initiative (NGI)/ NWO project nr. 050-060-810 and Research Institute for Diseases in the Elderly (RIDE). Abbas Dehghan is supported by a grant from NWO (Vici, 918-76-619).

The **SAS** study was funded by the British Heart Foundation.

The Swedish Research Council, the Swedish Heart & Lung Foundation and the Stockholm County Council (ALF) supported the **SHEEP** study.

**SMILE** was funded by the Netherlands Heart foundation (NHS 92345). Dr Rosendaal is a recipient of the Spinoza Award of the Netherlands Organisation for Scientific Research (NWO) which was used for part of this work.

The **Verona Heart Study** was funded by grants from the Italian Ministry of University and Research, the Veneto Region, and the Cariverona Foundation, Verona.

The **Atherosclerosis Risk in Communities** **Study** is carried out as a collaborative study supported by National Heart, Lung, and Blood Institute contracts N01-HC-55015, N01-HC-55016, N01-HC-55018, N01-HC-55019, N01-HC-55020, N01-HC-55021, and N01-HC-55022. The authors thank the staff and participants of the ARIC study for their important contributions.

The **KORA** (Kooperative Gesundheitsforschung in der Region Augsburg) research platform was initiated and financed by the Helmholtz Zentrum München - National Research Center for Environmental Health, which is funded by the German Federal Ministry of Education, Science, Research and Technology and by the State of Bavaria. Part of this work was financed by the German National Genome Research Network (NGFN-2 and NGFNPlus) and within the Munich Center of Health Sciences (MC Health) as part of LMUinnovativ.

Work described in this paper is part of the research portfolio supported by the Leicester NIHR Biomedical Research Unit in Cardiovascular Disease.

This work forms part of the research themes contributing to the translational research portfolio of Barts and the London Cardiovascular Biomedical Research Unit which is supported and funded by the National Institute of Health Research.

Disclosures of the CARDIoGRAM Consortium: Dr Absher reports receiving an NIH research grant for the ADVANCE study. Dr Assimes reports receiving an NIH research grant for the ADVANCE study. Dr Blankenberg reports receiving research grants from NGFNplus for Atherogenomics and from BMBF for CADomics. Dr Boerwinkle received research support from NIH/National Human Genome Research Institute (NHGRI), GWA for gene-environment interaction effects influencing CGD; NIH/NHLBI, Molecular epidemiology of essential hypertension; NIH/NHLBI, Genome-wide association for loci influencing coronary heart disease; NIH/NHLBI, Genetics of hypertension-associated treatment; NIH/NHLBI, Modeling DNA diversity in reverse cholesterol transport; NIH/NHLBI, 20-year changes in fitness and cardiovascular disease risk; NIH/NHLBI, Genetic epidemiology of sodium-lithium countertransport; NIH/National Institute of General Medical Sciences (NIGMS), Pharmacogenomic evaluation of antihypertensive responses; NIH/NIGMS, Genomic approaches to common chronic disease; NIH/NHLBI, Genes of the CYP450-derived eicosanoids in subclinical atherosclerosis; NIH/NHGRI-University of North Carolina, Chapel Hill, Genetic epidemiology of causal variants across the life course; and NIH/NHLBI, Building on GWAS for NHLBI-diseases: the CHARGE consortium. Dr Cupples reports receiving research grants from NIH/NHLBI, The Framingham Heart Study; NIH/NHLBI, Genome-wide association study of cardiac structure and function; NIH/NHLBI, Functional evaluation of GWAS loci for cardiovascular intermediate phenotypes; and NIH/NHLBI, Building on GWAS for NHLBI-diseases: the CHARGE consortium. Dr Halperin reports receiving research grants from NIH, subcontract Genome-wide association study of Non Hodgkin’s lymphoma; ISF, Efficient design and analysis of disease association studies; EU, consultant AtheroRemo; NSF, Methods for sequencing based associations; BSF, Searching for causal genetic variants in breast cancer and honoraria from Scripps Institute, UCLA. Dr Halperin also reports ownership interest in Navigenics. Dr Hengstenberg reports receiving research grants for EU Cardiogenics. Dr Holm reports receiving a research grant from NIH; providing expert witness consultation for the district court of Reykjavik; serving as member of the editorial board for decodeme, a service provided by deCODE Genetics; and employment with deCODE Genetics. Dr Li reports receiving research grant R01HG004517 and other research support in the form of coinvestigator on several NIH-funded grants and receiving honoraria from National Cancer Institute Division of Cancer Epidemiology and Genetics. Dr McPherson reports receiving research grants from Heart & Stroke Funds Ontario, CIHR, and CFI. Dr Rader reports receiving research grant support from GlaxoSmithKline. Dr Roberts reports receiving research grants from the Cystic Fibrosis Foundation, NIH, and Cancer Immunology and Hematology Branch; membership on the speakers bureau for AstraZeneca; receiving honoraria from Several; and serving as consultant/advisory board member for Celera. Dr Stewart reports receiving research grant support from CIHR, Genome-wide scan to identify coronary artery disease genes, and CIHR, Genetic basis of salt-sensitive hypertension in humans; other research support from CFI: Infrastructure support; and honoraria from the Institute for Biomedical Sciences, Academia Sinica, Taipei, Taiwan. Dr Thorleifsson is an employee of deCODE Genetics. Dr Thorsteinsdottir reports receiving research grants from NIH and EU; serving as an expert witness for a US trial; having stock options at deCODE Genetics; and having employment with deCODE Genetics. Dr Kathiresan reports receiving research grants from Pfizer, Discovery of type 2 diabetes genes, and Alnylam, Function of new lipid genes, and serving as consultant/advisory board member for DAIICHI SANKYO Merck. Dr Reilly reports receiving research grant support from GlaxoSmithKline. Dr Schunkert reports receiving research grants from the EU, project Cardiogenics; NGFN, project Atherogenomics; and CADnet BMBF. M. Preuss, L. Chen, and Drs König, Thompson, Erdmann, Hall, Laaksonen, März, Musunuru, Nelson, Burnett, Epstein, O’Donnell, Quertermous, Schillert, Stefansson, Voight, Wells, Ziegler, and Samani have no conflicts to disclose. Genotyping of PennCATH and MedStar was supported by Glaxo-SmithKline. Dawn M. Waterworth, Max C. Walker, and Vincent Mooser are employees of GlaxoSmithKline. PennCath/MedStar investigators acknowledge the support of Eliot Ohlstein, Dan Burns and Allen Roses at GlaxoSmithKline.

The funders had no role in study design, data collection and analysis, decision to publish, or preparation of the manuscript.

# Competing Interests

The following companies financially supported the CARDIoGRAM Consortium:
Boehringer Ingelheim, PHILIPS medical Systems, Sanofi/Aventis, Roche, Dade Behring/Siemens, AstraZeneca, GlaxoSmithKline, Berlin Chemie, Boots Healthcare, McNeil Pharma (former Woelm Pharma), MSD Sharp & Dohme,  Pfizer and  King Pharmaceuticals.

Dr Halperin also reports ownership interest in Navigenics. Dr Holm reports serving as member of the editorial board for decodeme, a service provided by deCODE Genetics and employment with deCODE Genetics. Dr Rader reports receiving research grant support from GlaxoSmithKline. Dr Roberts reports membership on the speakers bureau for AstraZeneca, receiving honoraria from Several; and serving as consultant/advisory board member for Celera. Dr Thorleifsson is an employee of deCODE Genetics. Dr Thorsteinsdottir reports serving as an expert witness for a US trial; having stock options at deCODE Genetics; and having employment with deCODE Genetics. Dr Kathiresan reports  receiving research grants from Pfizer, Discovery of type 2 diabetes genes, and Alnylam, Function of new lipid genes, and serving as consultant/advisory board member for DAIICHI SANKYO Merck. Dawn M. Waterworth, Max C. Walker, and Vincent Mooser are employees of GlaxoSmithKline.
There are no patents, products in development or marketed products to declare. This does not alter our adherence to all the PLoS ONE policies on sharing data and materials, as detailed online in the guide for authors.

M. Preuss, L. Chen, and Drs König, Thompson, Erdmann, Hall, Laaksonen, März, Musunuru, Nelson, Burnett, Epstein, O'Donnell, Quertermous, Schillert, Stefansson, Voight, Wells, Ziegler, and Samani have no conflicts to disclose.
