# Supplementary material for: RANTES/CCL5 and Risk for Coronary Events: Results from the MONICA/KORA Augsburg Case-Cohort, Athero-Express and CARDIoGRAM Studies
Source: PLoS One. 2011 Dec 6;6(12):e25734. doi: 10.1371/journal.pone.0025734 (PMC3232218; doi:10.1371/journal.pone.0025734)
Supplement: Table S1 — Baseline demographic, lifestyle and clinical characteristics of the study participants without and with incident coronary event during follow-up (MONICA/KORA Augsburg case-cohort study). (DOC) [file pone.0025734.s003.doc]

**Online Table S1.** Baseline demographic, lifestyle and clinical characteristics of the study participants without and with incident coronary event during follow-up (MONICA/KORA Augsburg case-cohort study).

| Variable | CHD cases | CHD non-cases | p ^b^ |
| --- | --- | --- | --- |
| n (women/men) | 83/280 | 932/976 | - |
| Age (yrs) ^a^ | 57.3 (0.4) | 52.3 (0.3) | <0.001 |
| Education <12 yrs | 78.0 | 76.9 | 0.669 |
| Body mass index (kg/m^2^) | 28.4 (0.2) | 27.1 (0.1) | <0.001 |
| Waist-to-hip ratio ^c^ | 0.930 (0.005) | 0.868 (0.002) | <0.001 |
| Systolic blood pressure (mm Hg) | 142.3 (1.1) | 133.6 (0.5) | <0.001 |
| Diastolic blood pressure (mm Hg) | 83.4 (0.6) | 81.5 (0.3) | 0.007 |
| History of hypertension (%) ^d^ | 65.8 | 41.4 | <0.001 |
| History of diabetes mellitus (%) | 17.9 | 4.9 | <0.001 |
| Total cholesterol (mg/dl) | 258.1 (2.6) | 237.1 (1.0) | <0.001 |
| LDL cholesterol (mg/dl) ^c^ | 170.6 (3.1) | 146.8 (1.2) | <0.001 |
| HDL cholesterol (mg/dl) | 49.2 (0.8) | 57.1 (0.4) | <0.001 |
| Ratio total cholesterol:HDL   cholesterol | 5.8 (0.1) | 4.5 (0.0) | <0.001 |
| C-reactive protein (mg/l) | 2.6 (1.1) | 1.4 (1.0) | <0.001 |
| Interleukin-6 (pg/ml) | 3.1 (1.1) | 2.0 (1.0) | <0.001 |
| RANTES (ng/ml) | 29.6 (0.8) | 28.0 (0.4) | 0.085 |
| Smoking status |  |  | <0.001 |
| Current smoker | 40.5 | 23.7 |  |
| Former smoker | 32.0 | 27.4 |  |
| Never smoker | 27.5 | 48.9 |  |
| Frequency of exercise |  |  | <0.001 |
| Active | 27.5 | 38.5 |  |
| Inactive | 72.5 | 61.5 |  |
| Alcohol consumption ^e^ |  |  | 0.169 |
| 0 g/d | 28.1 | 31.8 |  |
| >0-39.9/19.9 g/d | 41.3 | 42.2 |  |
| ≥40/20 g/d | 30.6 | 26.0 |  |
| Current hormone replacement   therapy ^f^ | 5.7 | 10.4 | 0.139 |
| Current use of oral contraceptives ^g^ | 0.0 | 13.5 | NA |
| Survey |  |  | <0.001 |
| S1 | 37.7 | 28.0 |  |
| S2 | 42.7 | 36.4 |  |
| S3 | 19.6 | 35.5 |  |

^a^ Data are weighted percentages for categorical variables, weighted means (standard errors) for normally distributed continuous variables and weighted geometric means (with antilog of standard errors of log means) for the skewed continuous variables C-reactive protein and interleukin-6.

^b^ Values from t test for continuous variables and from chi-square-test for categorical variables.

^c^ Only measured in participants of survey 2 and 3.

^d^ Defined as systolic blood pressure ≥140 mmHg and/or diastolic blood pressure ≥90 mmHg and/or use of antihypertensive medication given that subjects were aware that they had hypertension.

^e^ Men: 0, >0-39.9 g/d, ≥40 g/d; women: 0, >0-19.9 g/d, ≥20 g/d.

^f^ Only for women aged ≥50 yrs with no current use of oral contraceptives.

^g^ Only for women aged <50 yrs with no current hormone replacement therapy.

[Data have been reported before in Herder C, Illig T, Baumert J, Müller M, Klopp N, et al. (2008) Macrophage migration inhibitory factor (MIF) and risk for coronary heart disease: Results from the MONICA/KORA Augsburg case-cohort study, 1984-2002. Atherosclerosis 200: 380-388].
